# Posterior tibial slope increases over time in patients undergoing revision ACL reconstruction: A long‐term radiographic follow‐up study

**DOI:** 10.1002/ksa.12719

**Published:** 2025-07-01

**Authors:** Mahmut Enes Kayaalp, Jumpei Inoue, Koji Nukuto, Joseph D Giusto, Gillian Ahrendt, Jonathan D. Hughes, James J. Irrgang, Thorkell Snæbjörnsson, Jon Karlsson, Volker Musahl

**Affiliations:** ^1^ Department of Orthopaedic Surgery, UPMC Freddie Fu Sports Medicine Center University of Pittsburgh Pittsburgh Pennsylvania USA; ^2^ Department of Orthopaedics and Traumatology University of Health Sciences Istanbul Turkey; ^3^ Faculty of Health Sciences Brandenburg Brandenburg Medical School Theodor Fontane Brandenburg/Havel Germany; ^4^ Department of Physical Therapy, School of Health and Rehabilitation Sciences University of Pittsburgh Pittsburgh Pennsylvania USA; ^5^ Department of Orthopaedics, Institute of Clinical Sciences, Sahlgrenska Academy University of Gothenburg Gothenburg Sweden

**Keywords:** anterior cruciate ligament reconstruction, lower extremity deformities, meniscectomy, posterior tibial slope, radiography

## Abstract

**Purpose:**

Increased medial posterior tibial slope (PTS) is recognized as a significant risk factor for anterior cruciate ligament reconstruction (ACL‐R) failure. This study investigated radiographic changes in medial PTS over time among skeletally mature individuals undergoing revision ACL‐R and identified associated factors contributing to PTS changes.

**Methods:**

A 10‐year chart review of patients who underwent revision ACL‐R was performed. Inclusion criteria included having index knee radiographs spanning at least 5 years, with the first radiograph taken before primary ACL‐R, and age >14 years. Exclusion criteria included inadequate radiographs, multi‐ligament injuries and concomitant meniscus transplantation. Radiographic analysis involved determining the reliability of measuring PTS by two blinded raters across three time points using 25 selected radiographs, employing the standard error of measurement to ascertain the minimum detectable change (MDC), which was 1.0° (95% confidence interval). Changes in PTS were categorized into two groups based on the threshold of >2° (Group 1) and ≤2° (Group 2), to eliminate measurement errors. Two blinded researchers collected demographic data, clinical and operative histories and return‐to‐sports status.

**Results:**

Seventy‐six patients were included, with a mean age of 25.3 ± 10.3 years at the time of primary ACL‐R and an average radiographic follow‐up of 8.9 ± 3.6 years after the initial preoperative radiograph. Interobserver and intraobserver reliabilities indicated high consistency (Intraclass correlation [ICC] = 0.92 and 0.97–0.99, respectively). There was a statistically significant increase in PTS from initially measured to most recent radiographs, averaging an increase of 1.1 ± 1.5° (*p* < 0.001), surpassing the MDC. A PTS increase >2° was associated with previous posterior medial meniscal resection (*p* = 0.003). A significant association was observed between having a PTS ≥ 12° on the latest radiograph and a greater change in PTS over time (*p* < 0.001).

**Conclusions:**

Radiographic evaluations demonstrated a consistent increase in PTS over an average of 9 years among patients undergoing revision ACL‐R. Notably, a posterior medial meniscus resection was significantly linked to these increases. This continual increase in PTS may both contribute to and result from failed ACL‐R. PTS ≥ 12° may be considered a deformity that may continue to progress over time unless corrected.

**Level of Evidence:**

Level III.

**Abbreviations**
ACL: anterior cruciate ligament
ACL‐R: anterior cruciate ligament reconstruction
ICC: intraclass correlation coefficient
MDC: minimum detectable change
MRI: magnetic resonance imaging
PTS: posterior tibial slope
SD: standard deviation

## INTRODUCTION

Increased posterior tibial slope (PTS) has been recognized as a risk factor for failed anterior cruciate ligament reconstruction (ACL‐R) [29], and for failed revision ACL‐R [13, 33]. The impact of increased PTS on the ACL is attributed to increased anterior tibial translation [8], which causes an increase in ACL graft force [3]. Comparing ACL‐injured cohorts to healthy controls, clinical studies have consistently reported significantly higher mean PTS values in ACL‐injured patients [22, 27, 30]. Notably, outliers, defined as a radiographic PTS of ≥12° measured on a 15 cm segment of the proximal tibia, are more prevalent among ACL‐injured individuals [30]. Individuals undergoing multiple revision ACL‐Rs have shown even greater PTS compared to those undergoing a first‐time revision ACL‐R [2].

However, it remains unclear whether PTS undergoes progressive increases in ACL‐injured cohorts following physeal closure, similar to the progression seen in coronal malalignment [26]. This raises the possibility that high‐risk PTS (≥12°) reflects progressive deformity rather than natural variability, warranting investigation into whether PTS ≥ 12° is associated with greater increases over time compared to PTS < 12°.

The present study aimed to assess longitudinal changes in radiographic medial PTS among skeletally mature patients in a revision ACL‐R cohort and to identify factors associated with alterations in PTS. It was hypothesized that radiographic medial PTS would increase over time, and that patients with greater increases would be younger, have a longer duration of ACL deficiency, and have a higher rate of posterior meniscal injuries and/or history of meniscus resection. Additionally, patients with a greater PTS value on the most recent radiograph were expected to show a more pronounced increase in PTS over the follow‐up period.

This investigation is the first to evaluate true longitudinal changes in medial PTS using serial radiographs in a skeletally mature cohort undergoing revision ACL‐R. By assessing whether increased PTS represents a fixed anatomical risk factor or a progressive deformity, the study aims to provide clinically meaningful insights to inform decision‐making regarding the indication and timing of PTS‐reducing osteotomies.

## METHODS

Institutional review board approval was obtained prior to the start of the study (IRB: STUDY19030196). A chart review was conducted on a consecutive series of patients who underwent revision ACL‐R by seven high‐volume, fellowship‐trained sports medicine surgeons within one healthcare system, covering a 10‐year period up to October 2023. Inclusion criteria included patients who had undergone revision ACL‐R surgery, with index knee radiographs spanning at least 5 years—specifically, the first radiograph taken before the primary ACL‐R and any subsequent radiograph taken at least 5 years after the initial one. Exclusion criteria included inadequate or incomplete radiographs, multi‐ligament injuries, concomitant meniscus transplantation, prior history of infection and age ≤14 years at the time of first ACL‐R. An inadequate radiograph was defined as having less than 10 cm of the proximal tibia visible or an offset greater than 5 mm on the distal or posterior femoral condyles [4, 13, 21, 28, 33].

**Figure 1:**
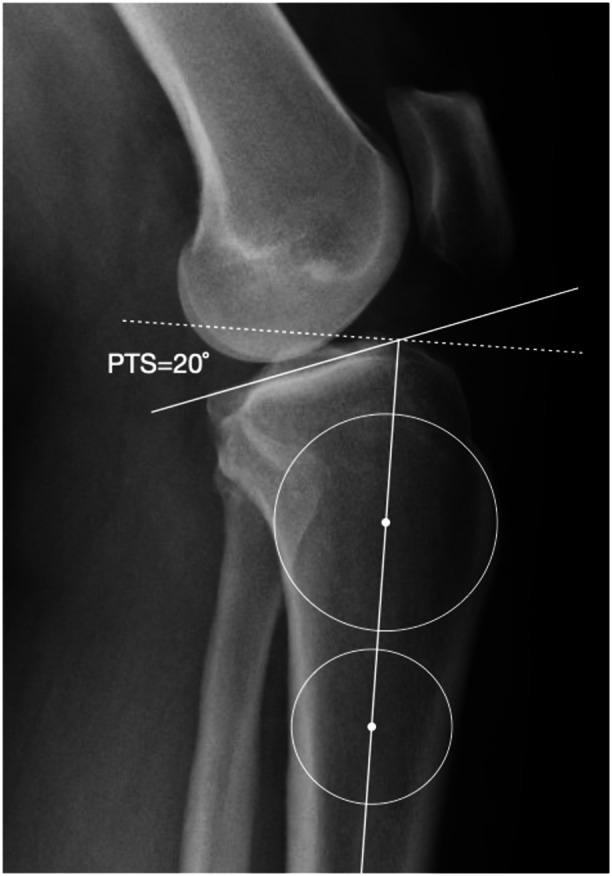
The figure illustrates posterior tibial slope (PTS) measurement on an adequate lateral knee radiograph from a 42‐year‐old female patient prior to anterior cruciate ligament (ACL) reconstruction. The radiograph is deemed adequate as it displays at least 10 cm of the tibia with less than 5 mm overlap of the femoral condyles distally or posteriorly. The PTS is measured using the proximal tibial axis, defined by a line connecting the centre points of two circles placed at the level of tibial tuberosity and 5 cm below it. The dashed white line represents the perpendicular line to the proximal tibial axis. The PTS is calculated as the angle between the dashed line and the line defining the concave surface of the tibial medial plateau. At the time of the ACL injury, the PTS measured 20°.

Radiographic PTS was measured as the angle between the medial tibial plateau and a line tangent to the proximal anatomic axis of the tibia (Figure 1). The widely accepted cutoff value of ≥12° for PTS was used for subsequent analysis [9, 25].

For intra‐ and inter‐rater reliability assessment, two raters independently measured 25 radiographs at three different time points, with one‐week intervals between each measurement. These 25 radiographs, consisting of both old and new radiographs from the study group, were selected using a random number generator.

**Figure 2:**
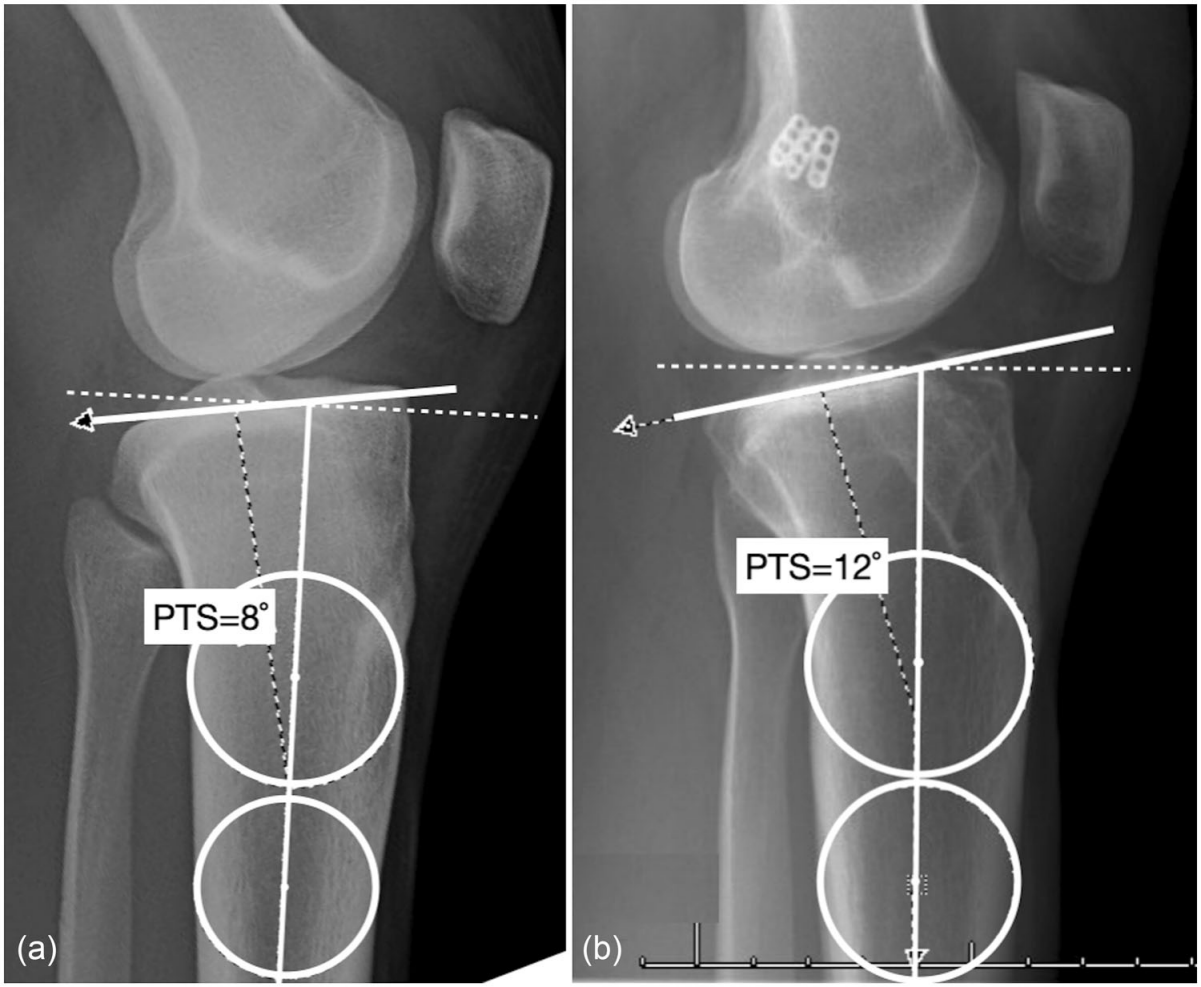
Figure (a) shows radiographic images of the right knee of a female patient at age 19 before her anterior cruciate ligament reconstruction (ACL‐R). Figure (b) displays a lateral knee radiograph of the same patient taken 7 years later, following her third revision ACL‐R. Three femoral side endobuttons are visible on (b). The posterior tibial slope increased from 8° at the initial presentation to 12° at the 7‐year follow‐up, after three ACL‐R procedures.

To account for PTS measurement error on radiographs, the standard error of measurement method was used to calculate the minimum detectable change (MDC) for PTS measurements on lateral knee radiographs, using the formula: 1.96 × (√2) × standard error of measurement (SEM), where SEM = SD × √(1 − ICC) (ICC: intraclass correlation coefficient) [32]. A total of 76 radiographs, a mix of old and new radiographs from the study patients, were measured blindly by three independent raters to determine the MDC with a 95% confidence interval (CI). The mean MDC across the three raters was calculated as 1.0°. Therefore, two patient groups were formed, with those exhibiting more than a 2° change in PTS classified as having a substantial change, free from measurement error (Figure 2). Group 1 consisted of patients with a PTS change of >2°, while Group 2 included those with a PTS change of ≤2°.

Two researchers, blinded to the measurements of PTS, conducted the chart review to collect demographic data and meniscal pathology‐related parameters. These parameters included the side of meniscal pathology (medial or lateral), the type of procedure (repair or resection), presence of chondral procedures, number of previous ipsilateral knee surgeries, number of ipsilateral ACL‐R surgeries, the duration of ACL deficiency in years throughout the follow‐up period including the time from injury to ACL‐R and from failure to revision ACL‐R. Baseline demographics, investigated surgical parameters and return‐to‐sport rates were then compared between the groups.

A PTS of ≥12° on the most recent radiograph and the increase in PTS between the earliest and latest radiographs were tested for a significant association.

### Statistical analysis

The statistical analysis of the data was conducted using the SPSS software (Version 25.0; IBM). Descriptive statistics were presented as numbers and percentages for categorical (discrete) data and as mean and standard deviation (SD) for continuous data. The normal distribution of the variables was examined using the coefficient of variation, histogram and Shapiro–Wilk test. The paired Wilcoxon test was used to compare the PTS measured at different time points. Pearson correlations were used to evaluate the relationships between the change in PTS across the entire group and the time elapsed between two radiographic assessments. The relationship between the groups and their demographic and clinical characteristics was examined using Pearson's Chi‐Square test with Yates' continuity correction. The Student's *t* test was used to analyze the association of the most recent PTS and the magnitude of PTS increase over time. Statistical significance was set at a priori as an alpha value of <0.05.

The study was powered to achieve 85% power with an alpha level of 0.05 and an effect size of 0.5 for PTS measurements on radiographs. Based on these parameters and an allocation ratio of 3, a total of 64 patients were deemed necessary: 16 in Group 1, exhibiting more than a 2° change in PTS, and 48 in Group 2, with a PTS change of ≤2°.

## RESULTS

**Figure 3:**
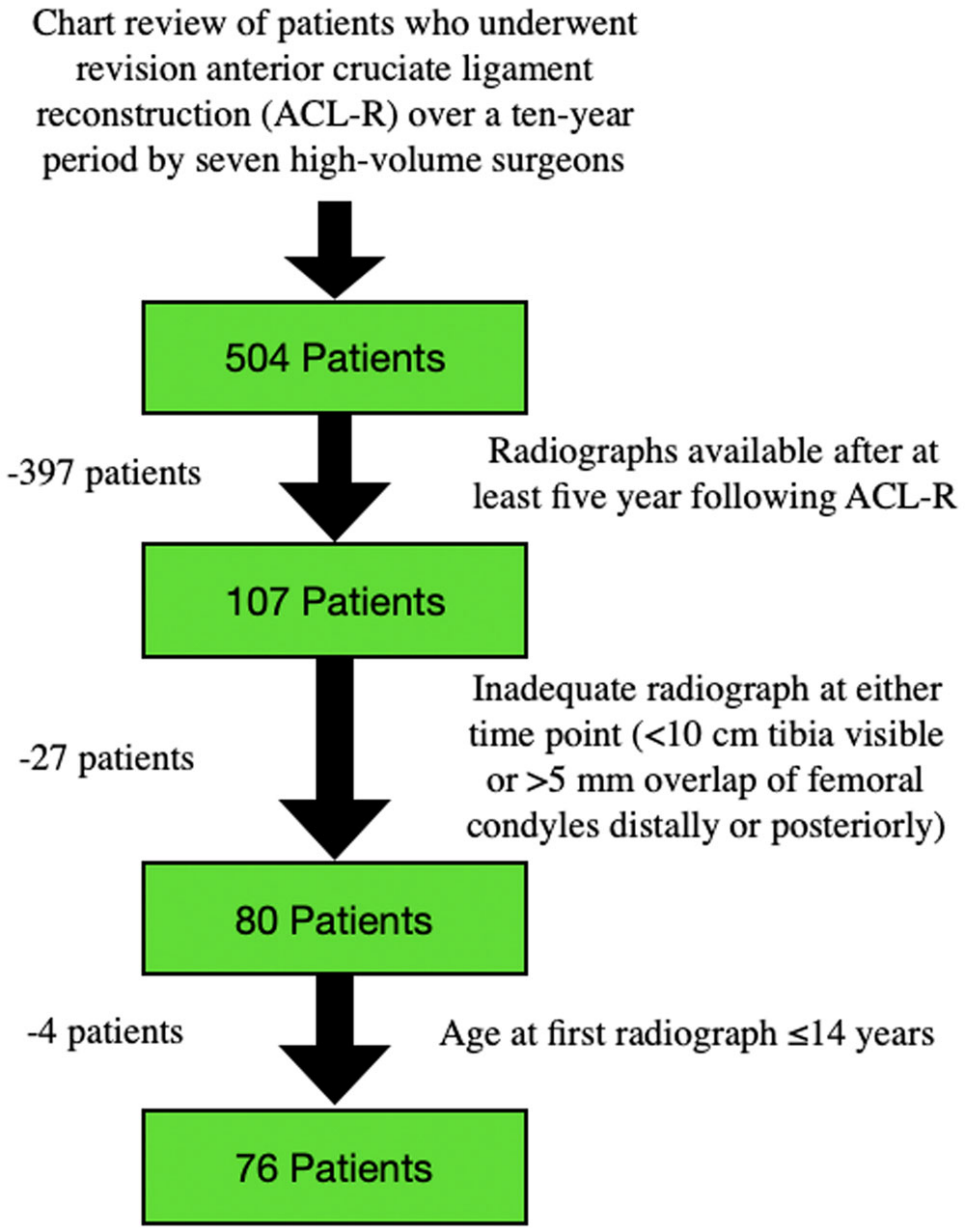
The figure presents the flowchart of the study cohort.

The preliminary search identified 504 patients. After applying the exclusion criteria, 76 patients with a mean age of 25.3 ± 10.3 years at the time of first ACL‐R were included in the analysis. The flowchart detailing the final patient selection is shown in Figure 3. The mean duration of radiographic follow‐up was 8.9 ± 3.6 years. Intraclass correlation coefficient (ICC) was 0.92 (95% CI: [0.79–0.97]). Intra‐observer reliability also demonstrated excellent consistency, with an ICC of 0.97 (95% CI: [0.93–0.99]) for Rater 1 and 0.99 (95% CI: [0.97–0.99]) for Rater 2.

There was a significant increase in PTS between the most recent and oldest available radiographs for the entire cohort, from 11.5 ± 3.2° to 12.6 ± 3.3°, respectively (*p* < 0.001). The mean increase in PTS was 1.1 ± 1.5°, surpassing the mean MDC of 1° (95% CI) across three raters. Group 1, consisting of patients with more than a 2° change in PTS, included 19 individuals, while Group 2, with a PTS change of ≤2°, included 57 patients.

The length of radiographic follow‐up did not correlate with the magnitude of PTS change. Group 1 (*n* = 19) did not differ from Group 2 (*n* = 57) in baseline demographics or other investigated parameters (Table 1). In the comparison of return to sports rates, 10 out of 14 patients in Group 1 (71%) and 31 out of 37 patients in Group 2 (84%) returned to sports, with no statistically significant difference between the groups (*p* = 0.894). However, the surgical history of posterior medial meniscal resection was significantly more frequent in Group 1 (58%) compared to Group 2 (25%) (p = 0.003) (Table 1). No other surgical variables were significantly associated with a greater change in PTS (n.s.).

**Table 1:** Demographics and comparison of parameters between the groups.

Parameter	Group 1 (>2° increase, *n* = 19), value ± standard deviation or value (min–max)	Group 2 (≤2° increase, *n* = 57), value ± standard deviation or value	*p* value
Age in years at the time of first ACL‐R (min–max)	22.4 ± 8 (15–45)	26.3 ± 10.9 (15–56)	0.099
Females, *n* (%)	8 (42%)	32 (56%)	0.633
Left side, *n* (%)	12 (63%)	28 (49%)	0.633
BMI in kg/m^2^	26.1 ± 6.3	25.8 ± 4.4	0.840
Radiographic follow‐up length in years	8.2 ± 3.6 (5–20)	9.1 ± 3.6 (5–18)	0.309
Number of previous ACL‐R	2.6 ± 0.8 (2–5)	2.4 ± 0.6 (2–4)	0.260
Number of ipsilateral previous knee surgeries	3.8 ± 1.5 (2–6)	3.8 ± 1.4 (2–8)	0.928
Duration of ACL deficiency in years (including the time from injury to ACL‐R and from failure to revision)	2.2 ± 3.5 (0.1–15)	2.6 ± 3.7 (0.1–22)	0.654
Posterior medial meniscal resection rate	11 (58%)	14 (25%)	0.003

Abbreviations: ACL, anterior cruciate ligament; ACL‐R, anterior cruciate ligament reconstruction; BMI, body mass index.

A PTS of ≥12° on the most recent radiograph was significantly associated with a greater increase in PTS between the most recent and oldest radiographs, with *p* value of <.001. Patients with a PTS of ≥12° showed a mean PTS increase of 1.6 ± 1.5°, compared to 0.3 ± 1.2° for those with a PTS of <12°.

## DISCUSSION

The most important finding of this study is the significant increase in radiographic medial PTS over time in individuals undergoing revision ACL‐R, with a mean follow‐up of 9 years and a minimum of 5 years. The mean PTS increased by 1.1° from the earliest to the most recent radiograph, with 25% of individuals exhibiting an increase greater than 2°. This progression in PTS was not correlated with the length of radiographic follow‐up nor other baseline demographic and clinical factors, except for a history of posterior medial meniscal resection, which was significantly more frequent in patients with an increase in PTS > 2°. These findings support the hypothesis that medial PTS may progressively increase in patients with ACL injuries, particularly in those with a history of posterior medial meniscal resection.

Previous studies have shown that revision ACL‐R patients have a greater mean PTS compared to those undergoing primary ACL‐R [2, 7]. The mean PTS increased with the number of revision ACL‐Rs [2]. However, the only previous longitudinal study on the same knees involved a limited number of adolescent patients [15]. That study found that the mean lateral PTS on magnetic resonance imaging (MRI) of ACL‐injured knees was 2° greater than in contralateral uninjured knees of the same patient, with a statistically significant increase of 0.9° in lateral PTS at a mean follow‐up of 10 years [15]. Despite numerous cross‐sectional studies examining PTS in various patient groups across different ages and sexes, whether healthy or ACL‐injured [19, 22, 31], the developmental pattern of sagittal knee alignment, including normal values and patterns of PTS change, remains largely unknown. In contrast, changes in coronal alignment parameters, such as joint space width, varus angle or hip–knee–ankle (HKA) angle, have been shown to vary over time in longitudinal follow‐up reports [6, 18, 26]. For example, joint space narrowing of 0.7 mm over 2 years [18], an increase in varus angle by 0.5° within 18 months [26], and an increase in HKA angle by 1.7° at a mean follow‐up of 2 years [6] have been previously described. The current study is the first to demonstrate a significant increase in PTS in the same knee over a mean radiographic follow‐up of 9 years.

The increase in PTS observed in this study after physeal closure may be driven by various pathophysiological pathways, each affecting different patient subgroups but resulting in a similar outcome—an increase in PTS. For instance, bone bruising at the time of ACL injury might influence subsequent changes in bony anatomy. Previous studies have shown that cartilage injuries often occur adjacent to areas of bone bruising following ACL injury and are accompanied by osteocyte necrosis in the subchondral bone [11]. Additionally, tibial posteromedial bone bruising has been found in 77% of patients at the time of ACL injury, making it the most common pattern [5]. Another study reported a significant association between bone bruising at the time of injury and progressive posttraumatic chondral disease with underlying bone reactive changes 5 years after ACL injury [14]. Furthermore, a prior study demonstrated that subchondral bone marrow lesions are highly associated with concomitant bone attrition in the same knee. This study identified subchondral bone marrow lesions as strong predictors of future attrition development, concluding that these lesions predispose to weakening of the subchondral osseous plate, causing detectable deformity or depression of the osseous articular contour [24]. Osteoarthritis progression after ACL‐R is a well‐known phenomenon, particularly after failed ACL‐R [1]. Notably, the osteoarthritis pattern of posteromedial erosions on the tibial plateau is mainly observed in ACL‐deficient knees [23]. This may result from femoral rollback on the tibial plateau in ACL‐deficient knees, as demonstrated in a previous study that performed a dynamic kinematic analysis under fluoroscopy using a two‐ to three‐dimensional registration technique [12]. The study found that the medial anteroposterior position of the femur in ACL‐deficient knees was significantly posterior compared to normal knees [12]. Taken together, these findings suggest that bone attrition in the form of depression of the posterior portion of the medial tibial plateau might underlie the increase in PTS observed in this study.

The current study confirmed that a history of posterior medial meniscal resection is associated with a >2° increase in PTS. Previous research suggests that changes in bony structures may result from a connected process, such as increased loading due to malalignment or damage to other protective joint structures like the menisci or intact articular cartilage [24]. Previous research also indicates that PTS has been associated with elevated forces on the posterior medial meniscal root [16], and a significant correlation exists between increased PTS and medial meniscus posterior horn tears [17]. Additionally, biomechanical theories propose that the ACL graft and menisci are interdependent in maintaining knee stability, relying on each other for this function [20]. This interdependence may also extend to bony morphology, particularly the PTS. Studies consistently show that increased anterior tibial translation [8] and greater forces on the posterior part of the meniscus are associated with an increased PTS [16]. A case‐control study demonstrated that tears in the medial meniscal posterior horn are linked to increased PTS [17]. According to the available literature, the significant association between posterior medial meniscal resection and a greater increase in PTS, therefore, seems plausible.

The last hypothesis of the current study was confirmed. It was found that a PTS of ≥12° on the most recent radiograph in a revision ACL‐R cohort was significantly associated with a greater increase in PTS between the most recent and oldest radiograph. This evidence supports the idea that outlier values, such as a PTS of ≥12°, result from a progressive deformity rather than natural variation. Previous studies have reported that revision ACL‐R cohorts exhibit greater PTS values compared to healthy controls or primary ACL‐R cases without failure after a follow‐up period of at least 2 years [2, 10, 30]. However, no prior study has identified the underlying causes for these differences.

This study has several limitations. First, the sample size is small, primarily due to the extensive inclusion and exclusion criteria. While the study is statistically powered to detect changes in radiographic PTS measurements, other conclusions related to surgical history may be underpowered. The 1‐week interval used between repeated measurements for intra‐rater reliability may have been too short to fully mitigate memory bias, and a longer interval, such as 6 weeks, would have been more appropriate. The study did not incorporate MRI‐based measurements or findings, nor did it include osteoarthritis‐related radiological classifications. Finally, the morphological cause of the change in PTS, particularly concerning deformity, was not analyzed in this study, as it was beyond its scope.

## CONCLUSION

Radiographic PTS increased in the cohort of individuals undergoing revision ACL‐R over an average follow‐up of 9 years. Among the surgical factors, posterior medial meniscus resection was the only one associated with a greater increase in PTS over time. A PTS of ≥12° on radiographs may indicate a more significant PTS increase in this cohort from the initial ACL injury to the most recent radiograph. These findings suggest that the increased PTS in the revision ACL‐R cohort may be more appropriately considered a deformity rather than a variability. Moreover, a PTS of ≥12° could be considered a progressive deformity that is likely to continue worsening over time if not surgically corrected.

## AUTHOR CONTRIBUTIONS

All listed authors have contributed substantially to this work. All authors have read and approved the final manuscript to be submitted and published. Conceptualization: Mahmut Enes Kayaalp, James J. Irrgang, Thorkell Snæbjörnsson, Jon Karlsson and Volker Musahl. Methodology: Mahmut Enes Kayaalp, Jumpei Inoue, Koji Nukuto, Joseph D Giusto, Gillian Ahrendt, Jonathan D. Hughes, James J. Irrgang, Thorkell Snæbjörnsson, Jon Karlsson and Volker Musahl. Formal analysis and investigation: Mahmut Enes Kayaalp, Jumpei Inoue, Koji Nukuto, Joseph D Giusto and Gillian Ahrendt. Writing—original draft preparation: Methodology: Mahmut Enes Kayaalp, Jumpei Inoue, Koji Nukuto, Joseph D Giusto, Gillian Ahrendt, Jonathan D. Hughes, James J. Irrgang, Thorkell Snæbjörnsson, Jon Karlsson and Volker Musahl. Writing—review and editing: Mahmut Enes Kayaalp, Jumpei Inoue, Jonathan D. Hughes, James J. Irrgang, Jon Karlsson and Volker Musahl. Funding acquisition: na. Resources: Mahmut Enes Kayaalp, Thorkell Snæbjörnsson, Jon Karlsson, Volker Musahl. Supervision: Mahmut Enes Kayaalp, James J. Irrgang, Thorkell Snæbjörnsson, Jon Karlsson and Volker Musahl.

## CONFLICT OF INTEREST STATEMENT

**Mahmut Enes Kayaalp**: Associate editor of *KSSTA*, co‐editor of *Joint Diseases and Related Surgery*, member of U‐45 Committee, *ESSKA*. **Jonathan D. Hughes**: Associate editor of *KSSTA*. **Jon Karlsson**: *Journal of ISAKOS* Board of Trustees, Deputy Chair. **Volker Musahl**: reports educational grants, consulting fees, and speaking fees from Smith & Nephew, educational grants from Arthrex and DePuy/Synthes, is a board member of the *International Society of Arthroscopy, Knee Surgery and Orthopaedic Sports Medicine (ISAKOS)*, and deputy editor‐in‐chief of *Knee Surgery, Sports Traumatology, Arthroscopy*. The remaining authors declare no conflicts of interest.

## ETHICS STATEMENT

Institutional Review Board approval was obtained prior to the start of the study (IRB: STUDY19030196).

## Data Availability

Available upon request.

## References

[1] Andrä K , Prill R , Kayaalp E , Irlenbusch L , Liesaus E , Trommer T , et al. Increase in cartilage degeneration in all knee compartments after failed ACL reconstruction at 4 years of follow‐up. J Orthop Traumatol. 2021;22:54. 34914026 10.1186/s10195-021-00618-3 PMC8677851

[2] Beel W , Schuster P , Michalski S , Mayer P , Schlumberger M , Hielscher L , et al. High prevalence of increased posterior tibial slope in ACL revision surgery demands a patient‐specific approach. Knee Surg Sports Traumatol Arthrosc. 2023;31:2974–2982. 36622421 10.1007/s00167-023-07313-2

[3] Bernhardson AS , Aman ZS , Dornan GJ , Kemler BR , Storaci HW , Brady AW , et al. Tibial slope and its effect on force in anterior cruciate ligament grafts: anterior cruciate ligament force increases linearly as posterior tibial slope increases. Am J Sports Med. 2019;47:296–302. 30640515 10.1177/0363546518820302

[4] Bixby EC , Tedesco LJ , Confino JE , Mueller JD , Redler LH . Effects of malpositioning of the knee on radiographic measurements: the influence of adduction, abduction, and malrotation on measured tibial slope. Orthop J Sports Med. 2023;11:23259671231164670. 37347024 10.1177/23259671231164670 PMC10280522

[5] Byrd JM , Colak C , Yalcin S , Winalski C , Briskin I , Farrow LD , et al. Posteromedial tibial bone bruise after anterior cruciate ligament injury: an MRI study of bone bruise patterns in 208 patients. Orthop J Sports Med. 2022;10:23259671221120636. 36276425 10.1177/23259671221120636 PMC9580091

[6] Colyn W , Bruckers L , Scheys L , Truijen J , Smeets K , Bellemans J . Changes in coronal knee‐alignment parameters during the osteoarthritis process in the varus knee. J ISAKOS. 2023;8:68–73. 36646170 10.1016/j.jisako.2022.12.002

[7] Duerr RA , Ormseth B , DiBartola A , Geers K , Kaeding CC , Siston R , et al. Association of elevated posterior tibial slope with revision anterior cruciate ligament graft failure in a matched cohort analysis. Am J Sports Med. 2023;51:38–48. 36412535 10.1177/03635465221134806

[8] Giffin JR , Vogrin TM , Zantop T , Woo SLY , Harner CD . Effects of increasing tibial slope on the biomechanics of the knee. Am J Sports Med. 2004;32:376–382. 14977661 10.1177/0363546503258880

[9] Goto K , Honda E , Iwaso H , Sameshima S , Inagawa M , Ishida Y , et al. Age under 20 years, pre‐operative participation in pivoting sports, and steep posterior tibial slope of more than 12 degrees are risk factors for graft failure after double‐bundle anterior cruciate ligament reconstruction. J Exp Orthop. 2024;11:e70102. 39629195 10.1002/jeo2.70102 PMC11612570

[10] Grassi A , Macchiarola L , Urrizola Barrientos F , Zicaro JP , Costa Paz M , Adravanti P , et al. Steep posterior tibial slope, anterior tibial subluxation, deep posterior lateral femoral condyle, and meniscal deficiency are common findings in multiple anterior cruciate ligament failures: an mri case‐control study. Am J Sports Med. 2019;47:285–295. 30657705 10.1177/0363546518823544

[11] Johnson DL , Urban Jr, WP , Caborn DNM , Vanarthos WJ , Carlson CS . Articular cartilage changes seen with magnetic resonance imaging‐detected bone bruises associated with acute anterior cruciate ligament rupture. Am J Sports Med. 1998;26:409–414. 9617404 10.1177/03635465980260031101

[12] Kage T , Taketomi S , Tomita T , Yamazaki T , Inui H , Yamagami R , et al. Anterior cruciate ligament‐deficient knee induces a posterior location of the femur in the medial compartment during squatting. J Orthop Res. 2023;41:1439–1448. 36484121 10.1002/jor.25501

[13] Kayaalp ME , Winkler P , Zsidai B , Lucidi GA , Runer A , Lott A , et al. Slope osteotomies in the setting of anterior cruciate ligament deficiency. J Bone Jt Surg. 2024;106:1615–1628. 10.2106/JBJS.23.01352 39066689

[14] Kia C , Cavanaugh Z , Gillis E , Dwyer C , Chadayammuri V , Muench LN , et al. Size of initial bone bruise predicts future lateral chondral degeneration in ACL injuries: a radiographic analysis. Orthop J Sports Med. 2020;8:2325967120916834. 32426411 10.1177/2325967120916834 PMC7222279

[15] Martin RK , Ekås GR , Benth J , Kennedy N , Moatshe G , Krych AJ , et al. Change in posterior tibial slope in skeletally immature patients with anterior cruciate ligament injury: a case series with a mean 9 years’ follow‐up. Am J Sports Med. 2021;49:1244–1250. 33683924 10.1177/0363546521997097

[16] Melugin HP , Brown JR , Hollenbeck JFM , Fossum BW , Whalen RJ , Ganokroj P , et al. Increased posterior tibial slope increases force on the posterior medial meniscus root. Am J Sports Med. 2023;51:3197–3203. 37715505 10.1177/03635465231195841

[17] Moon HS , Choi CH , Jung M , Lee DY , Eum KS , Kim SH . Medial meniscal posterior horn tears are associated with increased posterior tibial slope: a case‐control study. Am J Sports Med. 2020;48:1702–1710. 32407133 10.1177/0363546520917420

[18] Palmer JS , Monk AP , Hopewell S , Bayliss LE , Jackson W , Beard DJ , et al. Surgical interventions for symptomatic mild to moderate knee osteoarthritis. Cochrane Database Syst Rev. 2019;2019:CD012128. 10.1002/14651858.CD012128.pub2 PMC6639936 31322289

[19] Pangaud C , Laumonerie P , Dagneaux L , LiArno S , Wellings P , Faizan A , et al. Measurement of the posterior tibial slope depends on ethnicity, sex, and lower limb alignment: a computed tomography analysis of 378 healthy participants. Orthop J Sports Med. 2020;8:2325967119895258. 32047827 10.1177/2325967119895258 PMC6984458

[20] Papageorgiou CD , Gil JE , Kanamori A , Fenwick JA , Woo SLY , Fu FH . The biomechanical interdependence between the anterior cruciate ligament replacement graft and the medial meniscus. Am J Sports Med. 2001;29:226–231. 11292050 10.1177/03635465010290021801

[21] Peez C , Waider C , Deichsel A , Briese T , Palma Kries LK , Herbst E , et al. Proximal tibial anatomical axis and anterior tibial cortex‐based measurements of posterior tibial slope on lateral radiographs differ least from actual posterior tibial slope—a biomechanical study. J Exp Orthop. 2024;11:e70108. 39664925 10.1002/jeo2.70108 PMC11632255

[22] Pradhan P , Kaushal SG , Kocher MS , Kiapour AM . Development of anatomic risk factors for ACL injuries: a comparison between ACL‐injured knees and matched controls. Am J Sports Med. 2023;51:2267–2274. 37310177 10.1177/03635465231177465

[23] Raju PK , Kini SG , Verma A . Wear patterns of tibiofemoral articulation in osteoarthritic knees: analysis and review of literature. Arch Orthop Trauma Surg. 2012;132:1267–1271. 22622796 10.1007/s00402-012-1547-y

[24] Roemer FW , Neogi T , Nevitt MC , Felson DT , Zhu Y , Zhang Y , et al. Subchondral bone marrow lesions are highly associated with, and predict subchondral bone attrition longitudinally: the MOST study. Osteoarthritis Cartilage. 2010;18:47–53. 19769930 10.1016/j.joca.2009.08.018 PMC2818146

[25] Salmon LJ , Heath E , Akrawi H , Roe JP , Linklater J , Pinczewski LA . 20‐year outcomes of anterior cruciate ligament reconstruction with hamstring tendon autograft: the catastrophic effect of age and posterior tibial slope. Am J Sports Med. 2018;46:531–543. 29244525 10.1177/0363546517741497

[26] Sharma L . The role of knee alignment in disease progression and functional decline in knee osteoarthritis. JAMA. 2001;286:188–195. 11448282 10.1001/jama.286.2.188

[27] Tensho K , Kumaki D , Yoshida K , Shimodaira H , Horiuchi H , Takahashi J . Does posterior tibial slope laterality exist? A matched cohort study between ACL‐injured and non‐injured knees. J Exp Orthop. 2023;10:132. 38057689 10.1186/s40634-023-00702-z PMC10700254

[28] Vieider RP , Mehl J , Rab P , Brunner M , Schulz P , Rupp MC , et al. Malrotated lateral knee radiographs do not allow for a proper assessment of medial or lateral posterior tibial slope. Knee Surg Sports Traumatol Arthrosc. 2024;32:1462–1469. 38629758 10.1002/ksa.12170

[29] Webb JM , Salmon LJ , Leclerc E , Pinczewski LA , Roe JP . Posterior tibial slope and further anterior cruciate ligament injuries in the anterior cruciate ligament‐reconstructed patient. Am J Sports Med. 2013;41:2800–2804. 24036571 10.1177/0363546513503288

[30] Weiler A , Berndt R , Wagner M , Scheffler S , Schatka I , Gwinner C . Tibial slope on conventional lateral radiographs in anterior cruciate ligament‐injured and intact knees: mean value and outliers. Am J Sports Med. 2023;51:2285–2290. 37306059 10.1177/03635465231178292 PMC10353028

[31] Weinberg DS , Williamson DFK , Gebhart JJ , Knapik DM , Voos JE . Differences in medial and lateral posterior tibial slope: an osteological review of 1090 tibiae comparing age, sex, and race. Am J Sports Med. 2017;45:106–113. 27587744 10.1177/0363546516662449

[32] Weir JP . Quantifying test‐retest reliability using the intraclass correlation coefficient and the SEM. J Strength Cond Res. 2005;19:231–240. 15705040 10.1519/15184.1

[33] Winkler PW , Wagala NN , Hughes JD , Lesniak BP , Musahl V . A high tibial slope, allograft use, and poor patient‐reported outcome scores are associated with multiple ACL graft failures. Knee Surg Sports Traumatol Arthrosc. 2022;30:139–148. 33517476 10.1007/s00167-021-06460-8 PMC8800919

